# Latitudinal and meridional patterns of picophytoplankton variability are contrastingly associated with Ekman pumping and the warm pool in the tropical western Pacific

**DOI:** 10.1002/ece3.10589

**Published:** 2023-10-19

**Authors:** Yu Wang, Feng Zhao, Xuebao He, Weibo Wang, Lin Chang, Jianhua Kang

**Affiliations:** ^1^ Third Institute of Oceanography Ministry of Natural Resources Xiamen PR China; ^2^ Institute of Oceanology Chinese Academy of Sciences Qingdao PR China

**Keywords:** Ekman pumping, mesoscale eddy, picophytoplankton, spatial variability, tropical western Pacific, warm pool

## Abstract

Marine picophytoplankton plays a major role in marine cycling and energy conversion, and its effects on the carbon cycle and global climate change have been well documented. In this study, we investigated the response of picophytoplankton across a broad range of physicochemical conditions in two distinct regions of the tropical western Pacific. Our analysis considered the abundance, carbon biomass, size fraction, distribution, and regulatory factors of the picophytoplankton community, which included the cyanobacteria *Prochlorococcus* and *Synechococcus*, and small eukaryotic phytoplankton (picoeukaryotes). The first region was a latitudinal transect along the equator (142–163° E, 0° N), characterized by stratified oligotrophic conditions. The second region was a meridional transect (143° E, 0–22° N) known for its high‐nutrient and low‐chlorophyll (HNLC) conditions. Results showed that picophytoplankton contributed >80% of the chlorophyll *a* (Chl a), and was mainly distributed above 100 m. *Prochlorococcus* was the dominant organism in terms of cell abundance and estimated carbon biomass in both latitudinal and meridional transects, followed by *Synechococcus* and picoeukaryotes. In the warm pool, *Prochlorococcus* was primarily distributed below the isothermal layer, with the maximum subsurface abundance forming below it. The maximum *Synechococcus* abundance was restricted to the west‐warm pool, due to the high temperature, and the second‐highest *Synechococcus* abundance was associated with frontal interaction between the east‐warm pool and the westward advance of Middle East Pacific water. In contrast, picoeukaryotes formed a maximum subsurface abundance corresponding to the subsurface Chl *a* maximum. In the mixed HNLC waters, the cell abundance and biomass of the three picophytoplankton groups were slightly lower than those in the warm pool. Due to a cyclonic eddy, the contours of the maximum subsurface *Prochlorococcus* abundance were uplifted, evidently with a lower value than the surrounding water. *Synechococcus* abundance varied greatly in patches, forming a weakly high subsurface peak when the isothermal layer rose to the near‐surface (<50 m). The subsurface maximum picoeukaryote abundance was also highly consistent with that of the subsurface Chl *a* maximum. Correlation analysis and generalized additive models of environmental factors showed that nutrient availability had a two‐faceted role in regulating the spatial patterns of picophytoplankton in diverse latitudinal and meridional environments. We concluded through regression that temperature and light irradiance were the key determinants of picophytoplankton variability in the tropical western Pacific. This study provides insights into the changing picophytoplankton community structure with potential future changing hydroclimatic force.

## Graphical abstract

As part of the warmest region in WPWP, the tropical western Pacific Ocean is an ideal experimental site for the study of climate change and its effects on marine ecosystems. Physical processes regulated the picophytoplankton distribution patterns along broad latitudinal and meridional ranges in the tropical western Pacific.

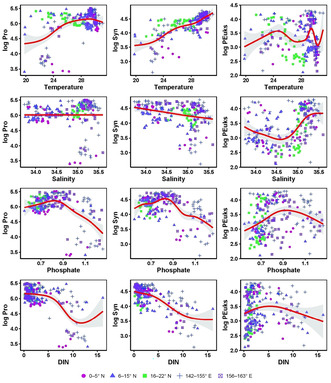

## 1 INTRODUCTION

The ecosystem of the tropical western Pacific, including the West Pacific warm pool (WPWP), is not uniform and can be separated into oligotrophic and mesotrophic waters (Hou et al., 2014; Messié & Radenac, 2006; Radenac et al., 2013). Mesotrophic waters of the Pacific cold tongue in the east, which are saline and have high‐nutrient (surface nitrate >2.8 μmol/L) and low‐chlorophyll contents (<0.1 mg/m^3^), are surrounded by oligotrophic waters of the warm pool to the west (Graham et al., 1987; Liu et al., 1997; Palmer & Mansfield, 1984). The WPWP is characterized by a sea‐surface temperature (SST) > 29°C, low surface salinity of <34, and oligotrophic conditions (Mackey et al., 1995; Messié & Radenac, 2006; Radenac & Rodier, 1996). Due to high precipitation, the halocline in the warm pool area is shallower than the thermocline, thus forming an obstacle layer (Lukas & Lindstrom, 1991) that inhibits nutrient transport from deeper water into the mixed layer, leaving the surface water depleted of nitrate (Lukas & Lindstrom, 1991; Mackey et al., 1995; Yoshikawa et al., 2006). However, Ekman pumping, a wind‐driven process that yields vertical velocities at the base of the Ekman layer and vertical nutrient fluxes (Pond & Pickard, 1983), is favorable for upwelling in the 7–9° N region of the western Pacific (Delcroix & Henin, 1989; Hou et al., 2014; Tournier, 1989). Therefore, compared with that in the surrounding latitudes, in the western Pacific, a local wind event can easily result in turbulent entrainment at the base of the mixed layer, allowing for nutrient input into the surface layer. In addition, the tropical western Pacific current has a banded structure, whereas that of the east–west current is staggered.

The smallest phytoplankton cells, which are most often categorized as picophytoplankton (0.2–2 μm), are the most abundant primary producers in the oceans. Composed of the cyanobacteria *Prochlorococcus* (Pro) (~0.8 μm) and *Synechococcus* (Syn) (~1 μm), and a polyphyletic group of picoeukaryotes (PEuks), picophytoplankton is responsible for 50%–90% of all primary production in open ocean ecosystems (Brewin et al., 2017; Li et al., 1983; Zubkov et al., 1998), playing a substantial role in maintaining the marine food web and contributing up to 30% of the total carbon exported to the deep ocean (Grob et al., 2007; Johnson & Lin, 2009; Richardson, 2019). Due to the importance of picophytoplankton, efforts have been made to incorporate picophytoplankton into dynamic ecosystem models to understand which environmental variables shape their biogeographic patterns (Buitenhuis et al., 2012; Le Quéré et al., 2005; Visintini et al., 2021; Wei et al., 2022). Much literature has recorded the importance of temperature on picophytoplankton abundance and distribution in various oceans (Agawin et al., 2000; Chen et al., 2014; Landry et al., 1996; Moisan et al., 2010). Wei et al. (2022) proposed that dissolved inorganic nitrogen (DIN) is a key determinant in driving large‐scale picophytoplankton variability. Moreover, light color acclimation is considered a vital process in the Syn distribution in the ocean (Grébert et al., 2018). On a global scale, niche models further identified photosynthetic active radiation (PAR), temperature, and nutrients as predictive variables for picophytoplankton abundance (Buitenhuis et al., 2012; Flombaum et al., 2013, 2020; Morán et al., 2010). Earth system models predicate that ocean phytoplankton biomass has decreased but that picophytoplankton biomass would increase in low‐latitude regions, being conducive to blooming of Pro (Flombaum et al., 2020). Subsequently, three datasets to assess the global picophytoplankton abundance were established to overcome the limitations of Earth system models (Visintini et al., 2021). Concerning physical processes, diverse physical conditions significantly influence the picophytoplankton distribution, causing dramatic changes in the distribution patterns both spatially and seasonally (Jiao et al., 2005; Lee et al., 2014; Ning et al., 2004, 2008; Šantić et al., 2011; Yuan et al., 2020). It was previously reported that picophytoplankton would dominate during the onset of anticyclonic eddies when the nitrate supply was inadequate due to their lower nutrient requirements compared to that of microphytoplankton; whereas, cyclonic eddies may lead to accumulation of large phytoplankton species in oligotrophic waters (Nishino et al., 2011; Vaillancourt et al., 2003). However, in tropical and subtropical oligotrophic waters, picophytoplankton could always dominate regardless of cyclonic or anticyclonic eddies (Casey et al., 2007; Chen et al., 2007). Long‐term observations indicated that cyclonic eddies in the developmental phase frequently showed higher chlorophyll *a* (Chl *a*) concentrations, with a higher proportion of Pro and a decrease in Syn abundance (Mcgillicuddy et al., 2007; Rii et al., 2008). Correspondingly, during the recessional phase, the Chl *a* concentration in the center of the cyclonic eddies decreased significantly, and the edges of the cyclonic eddies began to show higher biomass and productivity with an increase in Syn abundance (Mcgillicuddy et al., 2007; Rii et al., 2008). Oceanic fronts are widespread features that separate distinct water masses and are well known to control the distribution of microbial communities (Baltar & Arístegui, 2017; Clayton et al., 2014). Permanent fronts, such as the Azores Front and Kuroshio Extension, can act as ecological boundaries in the ocean in terms of microbial community structure and biogeochemical cycling (Baltar & Arístegui, 2017; Clayton et al., 2014). Frontal circulations generate vigorous vertical motions (Rudnick & Luyten, 1996), which are important in driving the supply of inorganic nutrients from below the mixed layer and in modulating the light environment for phytoplankton (Baltar & Arístegui, 2017; Clayton et al., 2014; Nagai et al., 2012; Radenac et al., 2013). Thus, the distribution of picophytoplankton groups and their responses to physical processes, particularly the different stages of eddies, has been examined in‐depth and is attracting increasing attention (Blanchot et al., 2001; Brown et al., 2008; Chen et al., 2015; Lévy et al., 2009; Ning et al., 2004, 2008; Ribeiro et al., 2016).

Under the various global warming scenarios, the picophytoplankton contribution relative to large phytoplankton will increase in the strongly stratified upper ocean (Buitenhuis et al., 2012; Coello‐Camba & Agustí, 2021; Morán et al., 2010). Studies have inferred that future warmer ocean conditions may lead to elevated biomass in regions that were already dominated by picophytoplankton (Flombaum et al., 2020). A high Pro abundance has been reported along with various strains and genomes in the tropical western Pacific (Biller et al., 2014; Kelly et al., 2012; Wang, Su, et al., 2022; Wang, Wei, et al., 2022). With global warming, the Pro abundance may increase by 29% by the end of this century, and its distribution trend may expand to the north and south poles (Flombaum et al., 2013, 2020). Consequently, the changes experienced by picophytoplankton due to climate change will significantly impact the global ocean ecosystem and biogeochemical cycles (Coello‐Camba & Agustí, 2021; Flombaum et al., 2013). Hence, as part of the warmest region in the WPWP, the tropical western Pacific Ocean is an ideal experimental site for studying climate change and its effects on marine ecosystems (Rowe et al., 2011). In the present study, we hypothesized that the picophytoplankton community structure in the tropical western Pacific may display dramatic spatial variations in response to diverse physical conditions under the context of global climate change. Specifically, physical processes regulate the picophytoplankton community structure and biogeochemical roles by redistributing light and nutrient availability in the water column. In this study, a flow cytometric analysis of picophytoplankton was performed along two transects in the tropical western Pacific to determine: (1) the abundance, carbon biomass, and distribution of Pro, Syn, and PEuks along latitudinal and meridional patterns; and (2) the way in which the picophytoplankton community structure varied in response to geographical conditions and hydrological regimes from the equator to the subtropics. Our results provide a reference for the picophytoplankton community on a large scale in this chronically under‐sampled region.

## 2 MATERIALS AND METHODS

### 2.1 Observation transects

**FIGURE 1:**
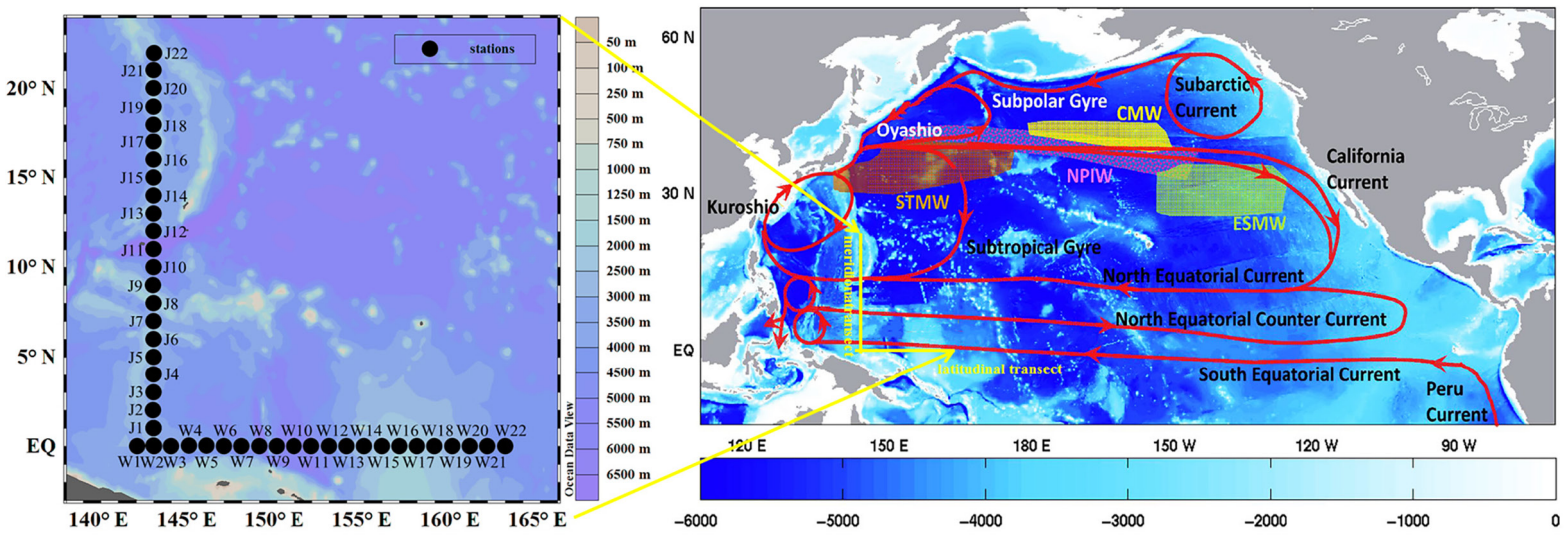
Location of sampling station in the tropical western Pacific Ocean during May 2017. The schematic of the Pacific surface circulation and water mass distribution is modified from Tseng et al., 2016. The most important water masses are NPIW, North Pacific Intermediate Water; CMW, Central Mode Water; ESMW, Eastern Subtropical Mode Water; and STMW, Subtropical Mode Water.

The study area and transects spanned the tropical western Pacific, and the current systems included the North Equatorial Current, North Equatorial Countercurrent, coastal current of Papua New Guinea, and subsurface currents (such as the equatorial undercurrent). The meridional transect was located at 143° E, spanning between 0 and 22 latitudes. The latitudinal transect was located on the equator, across the west Pacific warm pool, spanning between 142 and 163 longitudes (Figure 1). There were 22 stations on each transect.

### 2.2 Sampling and data collection

Seawater sampling and temperature and salinity measurements were performed using a SeaBird conductivity/temperature/depth meter (SEB‐911 plus, FL, USA) equipped with 12‐L Niskin bottles. Flow cytometry samples (6 mL) for picophytoplankton were collected from six depths (3, 30, 75, 100, and 150 m) at most stations and from the subsurface chlorophyll maximum layer. Nutrients and other oceanographic variables were collected at a 300 m depth.

Total Chl *a* was measured from samples collected at the same depth as picophytoplankton by filtering 2 L of seawater through Whatman GF/F filters (diameter 47 mm × 0.45 μm). To determine the contribution of picophytoplankton to total Chl *a*, the size fraction of Chl *a* was measured at the same station and dates as the picophytoplankton. Size‐fractionated Chl *a* concentrations were measured in 1500 mL water samples that were filtered sequentially through 20 μm nylon sieves for microphytoplankton, 2 μm polycarbonate membranes for nanophytoplankton, and 0.2 μm Whatman GF/F filters for picophytoplankton. All filters were stored at −80°C until analysis. In the laboratory, Chl *a* was extracted with 90% acetone for 24 h in the darkness, and the Chl *a* concentration was then analyzed using a 10‐AU Turner Design fluorometer (Turner‐Designs‐Model 10) as previously described by Parsons et al. (1984).

Water samples for nutrient analysis were filtered through 0.45‐μm cellulose–acetate filters and stored at −20°C. Nutrient concentrations were determined using a QuAAtro nutrient automatic analyzer (Bran+Luebbe Gmbh, Germany) according to classical colorimetric methods (Hansen & Koroleff, 1999). The detection limit was 0.01 μmol/L for nitrate and nitrite, 0.03 μmol/L for ammonium, and 0.02 μmol/L for phosphate and silicic acid. According to the methods described in “Specifications for oceanographic survey‐part 4: survey of chemical parameters in seawater” (General Administration of Quality Supervision (GAQS), 2008), total organic carbon (TOC) in seawater was determined via the combustion oxidation‐non‐dispersive infrared absorption method. Dissolved oxygen (DO) was determined using iodometry. pH was determined using the mercuric chloride oxidation method.

Seawater samples (6 mL) for flow cytometric analysis were fixed with 1% paraformaldehyde and 0.5% glutaraldehyde (final concentration) for 15 min at room temperature (25°C) and flash frozen in liquid nitrogen. In the laboratory, picophytoplankton enumeration was performed using a BD FACS Calibur flow cytometer (Becton‐Dickinson Accuri C6, USA) equipped with a 488 nm argon laser according to Marie et al. (2000) (Figure S1). Fluorescent beads (2 μm; Polysciences) were added to 1 mL replicated samples as the instrument internal standard (Jiao et al., 2005). Picophytoplankton biomass was estimated based on the carbon conversion coefficients of various groups, which for Syn, Pro, and PEuks were 250, 53, and 964 fgC/cell, respectively (Wei et al., 2020). Picophytoplankton abundances were depth‐averaged over the top 150 m following integration using a trapezoid rule (expressed as I_Pro_, I_Syn_, and I_PEuks_).

The merged sea surface Chl *a* and euphotic depth (Z_eu_; depth at which the down‐welling irradiance is 1% of its surface value) data were retrieved from the Globcolour website (http://hermes.acri.fr/). The isothermal depth (Z_T_) is defined as the depth at which the temperature differs by 0.8°C from the 10 m reference value (Levitus et al., 2000). The ratio of the euphotic depth (Z_eu_) to the isothermal depth (Z_T_) was used as an index of light availability above the thermocline. In addition, WindSat SST of 1/4 degree daily optimum interpolation (APDRC, https://apdrc.soest.hawaii.edu/) and SMAP sea‐surface salinity Level 2C orbital data (http://data.remss.com/smap/) were used to identify the general hydrological features. The merged sea level anomaly (SLA) at a spatial resolution of 1/4 degree and a time resolution of 1 day (ftp://ftp.aviso.oceanobs.com/global/) and ocean surface current analysis data (http://www.oscar.naaa.gov) at 5‐day intervals and 1 × 1 spatial resolution were extracted to identify the main currents and mesoscale eddies. Wind stress curl and Ekman pumping velocity (EPV) based on ASCAT wind vectors were coagulated according to Stewart (2004) and Wang, Su, et al. (2022); Wang, Wei, et al. (2022). The gridded averaged wind at 10 m above mean sea level was retrieved from the ASCAT scatterometer onboard the Metop satellites, and had spatial resolutions of 0.25° in longitude and latitude (ftp://ftp.ifremer.fr/ifremer/cersat).

### 2.3 Data analysis

Picophytoplankton abundances in each layer were log10‐transformed to improve the normality for statistical analysis. Statistical significance was set at *p* < .05 in all tests. Depth‐integrated variables were calculated by dividing the trapezoidal integration of measured values for each variable by the maximum sampling depth. These depth‐integrated variables, which involve a series of abundance variations with depth, were better suited for our distributional patterns than discrete sample data. Two kinds of statistical methods were applied to analyze the relationship between the picophytoplankton community structure and environmental factors. Redundancy analysis (R v4.0.2) was carried out to elucidate the interrelations between the biological group (picophytoplankton community structure from the two transects) and their corresponding environmental group (environmental factors including temperature, salinity, pH, DO, TOC, Z_eu_/Z_t_, nutrients, and Chl *a*), which identified the key environmental factors contributing significantly to the total variance of the survey area. Subsequently, a series of generalized additive models (GAMs) were applied to fit the responses of the three picophytoplankton groups to key environmental factors (R v4.0.2).

## 3 RESULTS

### 3.1 Physical processes determined by remote sensing

**FIGURE 2:**
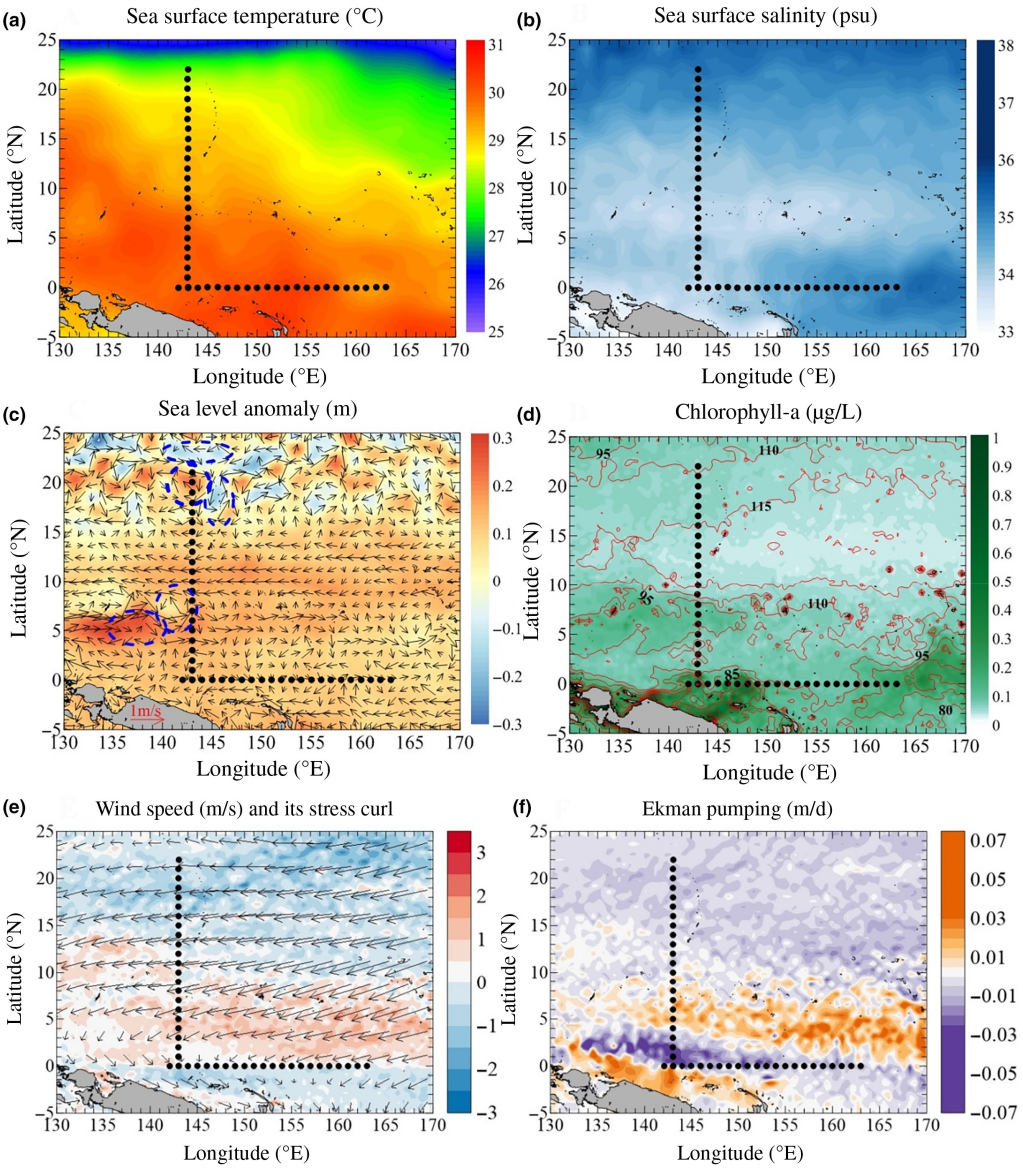
Sampling stations (black dots) overlaid on (a) sea surface temperature, (b) sea surface salinity, (c) sea level anomaly overlaid by sea surface current, blue circles indicated eddies. (d) sea surface chlorophyll a with euphotic depth (red contour lines), (e) wind speed and stress curl, (f) Ekman pumping velocity in the tropical western Pacific Ocean corresponding to the sampling period during May 2017.

Remote sensing observations (Figure 2) showed that the warm pool in the tropical western Pacific was characterized by an SST > 29°C and low surface salinity (<34). The cold water (<29°C) was located northeast of 10° N. A saline tongue was observed east of 155° E, with more saline water above 15° N. One large anticyclonic eddy with a deeper Z_eu_ (95–110 m) extended within 130–142° E, 4–7° N. At a nearby location, two cyclonic eddies were confined to 6° N and 7° N, thereby influencing adjacent stations. However, there were many more cyclonic and anticyclonic eddies above 15° N. One station, J22, at the northernmost station, was found to be at the edge of a cyclonic eddy. Furthermore, anticyclonic eddies were recorded at three northernmost stations, including J21, J20, and J19. The surface Chl *a* concentration was generally <0.10 mg/m^3^, except for some patches adjacent to land in which it increased in both the western and eastern ends along the meridional transect on the equator. Generally, Z_eu_ ranged 65–118 m. Significant Ekman pumping caused by the wind stress curl was located at approximately 140–165° E, 6–15° N, and the strong EPV area was approximately consistent with that of the location of the high Chl *a* concentration (Figure 2).

### 3.2 Oceanographic conditions

**FIGURE 3:**
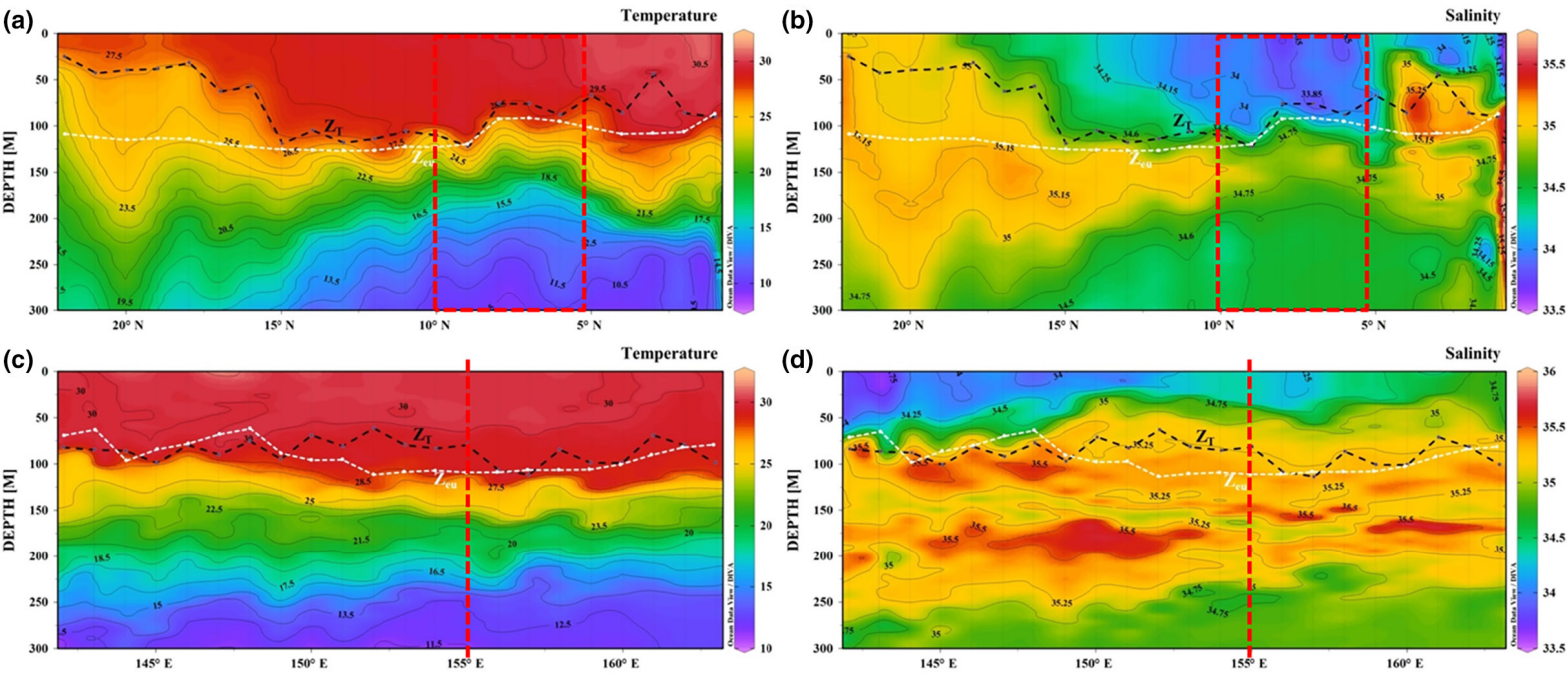
Profiles of temperature and salinity along the (a, b) meridional and (c, d) latitudinal transects. The dashed dark line represents isothermal depth. The dashed white line represents euphotic depth.

The temperature isolines fluctuated greatly along the meridional transect (Figure 3a,b) and the isothermal depth was between 25 and 120 m. Taking 10° N as the boundary, the isothermal depth had an obvious concave structure at both ends. The salinity contour also fluctuated greatly, which may have been associated with the water exchange between the northern and southern hemispheres. Near the equator (1° S–5° N), the core salinity of subsurface water was >35.3 with the maximum appearing at 200 m. The halocline ranged 50–100 m. In the North Equatorial current area (10–20° N), the maximum salinity was approximately 35.1 near 150 m, and the minimum was approximately 34.2 at 300 m. The North Pacific intermediate water was located at 200 m. In contrast, the isotherm fluctuated slightly along the latitudinal transect (Figure 3c,d). Overall, the isotherms above 200 m tended to decrease from west to east. Warm water (28°C) was above 100 m. A thermocline was observed between 100 and 150 m, with the temperature below gradually decreasing to 14°C at 300 m. Surface salinity was relatively low, generally >35 between 100 and 250 m, which is a characteristic of subtropical high salinity waters in the north–south sphere. There was a multi‐core structure in the high‐salinity center, which may have resulted from the reverse flow of the equatorial subsurface current and surface South Equatorial current. Minimum salinity occurred at 300 m where the South Pacific intermediate water was present.

**FIGURE 4:**
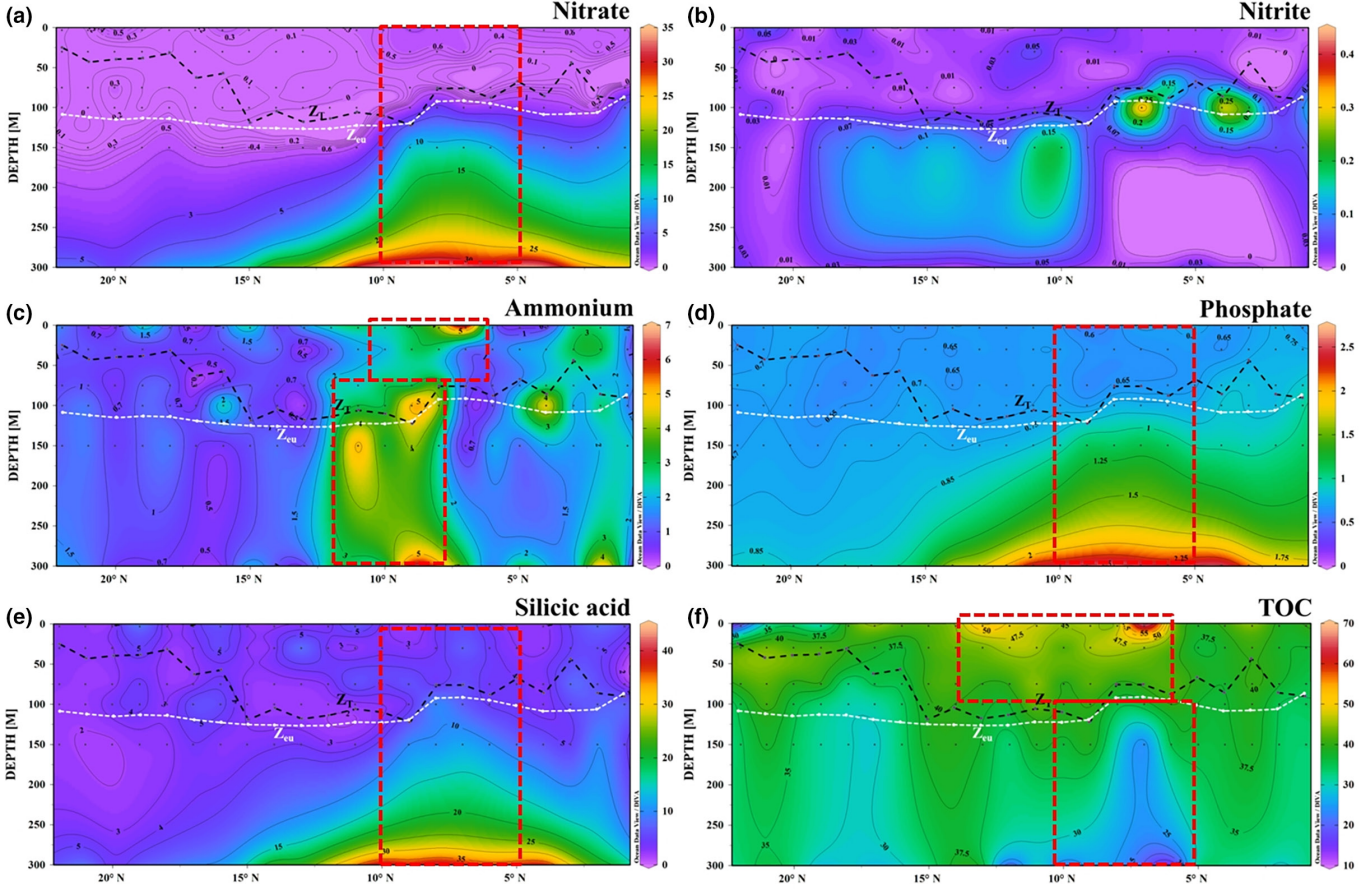
Meridional profiles of nutrients concentrations: (μmol/L) (a) nitrate, (b) nitrite, (c) ammonium, (d) phosphate, (e) silicic acid, and (f) TOC concentrations (mg/m^3^). The dashed dark line represents isothermal depth. The dashed white line represents euphotic depth. The dashed red line suggests Ekman pumping induced upwelling and its related fluctuation in euphotic zone.

In the meridional transect, the surface nitrate, nitrite, and phosphate concentrations at some stations were below the detection limits (Figure 4a,b,d). The maximum nitrate, phosphate, and silicic acid concentrations reached 34.57, 2.56, and 40.39 μmol/L, respectively. The overall nitrite content in this transect was <1 μmol/L in most areas. There was strong Ekman pumping‐induced upwelling from the high‐nutrient North Pacific intermediate water in the 5–12° N range was affected by Ekman pumping. The upwelling water contained nitrate concentrations up to 34.57 μmol/L with an N:P ratio of approximately 16:1, similar to the normal Redfield ratio. The ammonium concentration in the upper euphotic region of the upwelling center (7–9° N) was >5 μmol/L. The upwelling water was characterized by low TOC, with an isobath of 28 μmol/L reaching 150 m (Figure 4f). The rich nitrate and phosphate brought by the upwelling water provided an important nutritional basis for biological activity in the upper euphotic layer, inducing higher TOC in the euphotic zone (<100 m) than that in the surrounding areas.

**FIGURE 5:**
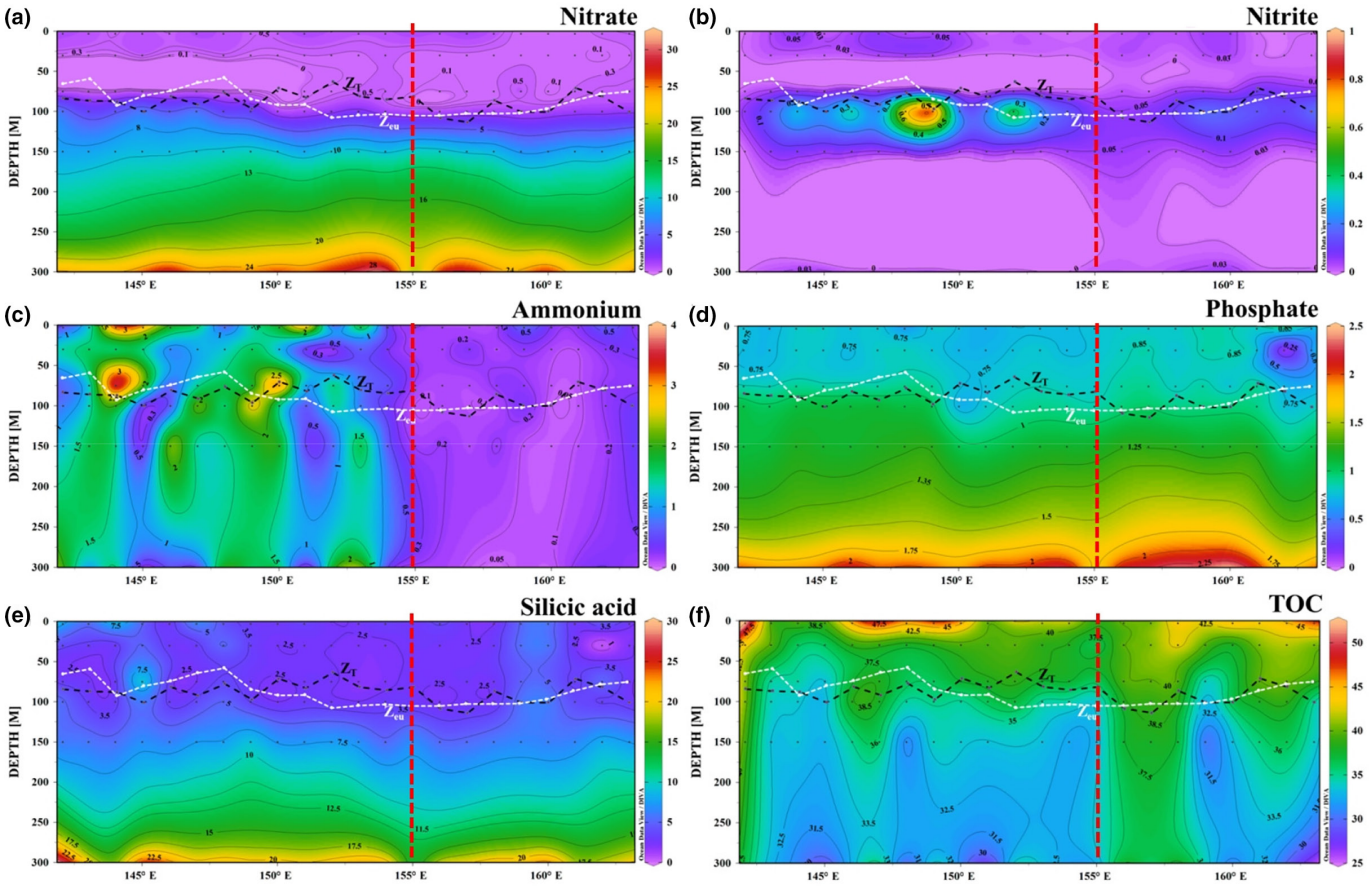
Latitudinal profiles of nutrient concentrations: (μmol/L) (a) nitrate, (b) nitrite, (c) ammonium, (d) phosphate, (e) silicic acid, and (f) TOC concentrations (mg/m^3^). The dashed dark line represents isothermal depth. The dashed white line represents euphotic depth. The dashed red line suggests the boundary of the frontal interaction in the warm pool.

Along the latitudinal transect, the nitrate, phosphate, and silicic acid concentrations increased with depth, in line with the characteristics of South Pacific water (Figure 5a,d,e). The maximum nitrate and phosphate concentrations reached 31.36 and 2.47 μmol/L, respectively, and the silicic acid concentration ranged 2.04–27.94 μmol/L. High Si:N (~0.86:1) and low N:P (~13:1) were located below 200 m east of the nutrient contour at 156° E. This indicated that more nitrate than phosphate was consumed in the vertical upward transportation of deep water. The nitrite concentration in this transect ranged 0.01–0.96 μmol/L. The ammonium concentration ranged 0.01–3.85 μmol/L; it was <0.2 μmol/L east of 156° E and significantly lower in the west. Corresponding to the consumption of main nitrate (low N:P), the TOC contour of 38.5 μmol/L at 200 m in the east of 156° E sunk, and was higher than that in the west (Figure 5f).

Overall, the vertical distribution trends of nitrate, phosphate, and silicic acid in the tropical western Pacific were similar (Figures 4 and 5). The nutrient content was low at 100 m, with an obvious vertical stratification below it, and gradually increased with depth. The nitrite concentration was low at 75 m and below 150 m, with the maximum near 100 m.

### 3.3 Chl *a* and picophytoplankton size fraction

**FIGURE 6:**
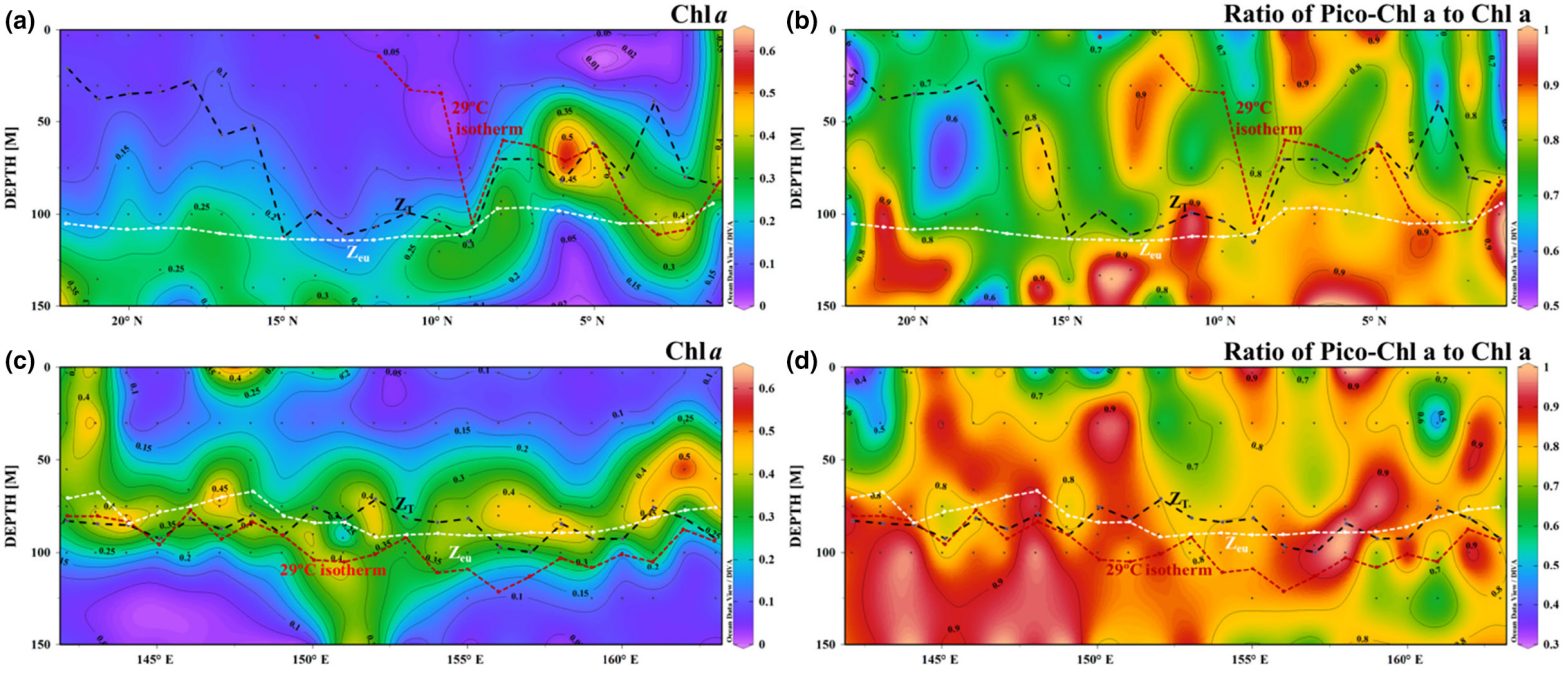
Distributions of total Chl *a* concentrations (μg/L) and the contribution of picophytoplankton to total biomass (Pico%) along the (a, b) meridional and (c, d) latitudinal transects. The dashed dark line represents isothermal depth. The dashed white line represents euphotic depth. The dashed dark red line represents the 29°C isotherm.

According to the raw data, the Chl *a* concentration was low in the surface layer, reaching 0.02–0.35 μg/L to 30 m. The concentration was 0.25–0.62 μg/L at 75–100 m, and it gradually decreased to 0.05 μg/L at 150 m. The maximum Chl *a* concentration at J8 was observed at 75 m. The subsurface Chl *a* maximum (SCM) distribution differed between the transects (Figure 6a,c). SCM in the meridional transect changed greatly with a decrease in latitude, generally rising from 150 to 75 m. SCM in the latitudinal transect was mostly retained at 75 m, with the maximum value observed at the surface near the warm pool (Figure 6b). The depth‐integrated Chl *a* concentration was 0.19 ± 0.05 μg/L (*n* = 44) ranging 0.10–0.31 μg/L (Table 1). The 0.2‐μm size fraction of Chl *a* revealed that picophytoplankton accounted for >79% (meridional transect) and >80% (latitudinal transect) of the total Chl *a* in the upper 150 m.

**TABLE 1:** The depth‐integrated Chl *a* concentrations (μg/L), picophytoplankton abundances (cells/mL), and ratio of Pro:Syn in the tropical western Pacific Ocean.

Regions	Chl *a*	Pro	Syn	PEuks	Ratio of pro:Syn
Meridional Transect	0.16 ± 0.04 (0.10–0.28)	144,869 ± 18,562 (116,734–177,249)	21,607 ± 4346 (13160–30,219)	4576 ± 1494 (2075–7719)	8.29 ± 2.06 (4.89–12.58)
Latitudinal Transect	0.22 ± 0.04 (0.17–0.31)	163,300 ± 22,740 (125,282–222,478)	32,378 ± 5452 (22391–40,318)	6714 ± 2901 (2530–11,858)	5.53 ± 0.88 (3.82–7.15)
Entire Area	0.19 ± 0.05 (0.10–0.31)	154,084 ± 22,532 (116,734–222,478)	22,993 ± 7309 (13,160–40,318)	5645 ± 2524 (2075–11,858)	6.91 ± 2.10 (0.10–0.31)

*Note*: Minimal, maximal, and average values are presented.

Ekman pumping transported North Pacific intermediate water with nitrate concentrations >20 μmol/L and phosphate concentrations >1.5 μmol/L to the upper euphotic zone between 200 m (Figure 4). The nitrate and phosphate brought by the upwelling water were gradually consumed by phytoplankton in the upper euphotic layer, with the nitrate consumption rate being significantly higher than that of phosphate, since the N:P was <16. With nitrate and phosphate consumption, phytoplankton proliferated in the upper euphotic layer, resulting in a 5‐fold spike in the Chl *a* concentration (7–9° N) in the upwelling center compared to that in the non‐upwelling area, reaching 0.5 mg/m^3^ (Figure 6a).

### 3.4 Picophytoplankton abundance

**FIGURE 7:**
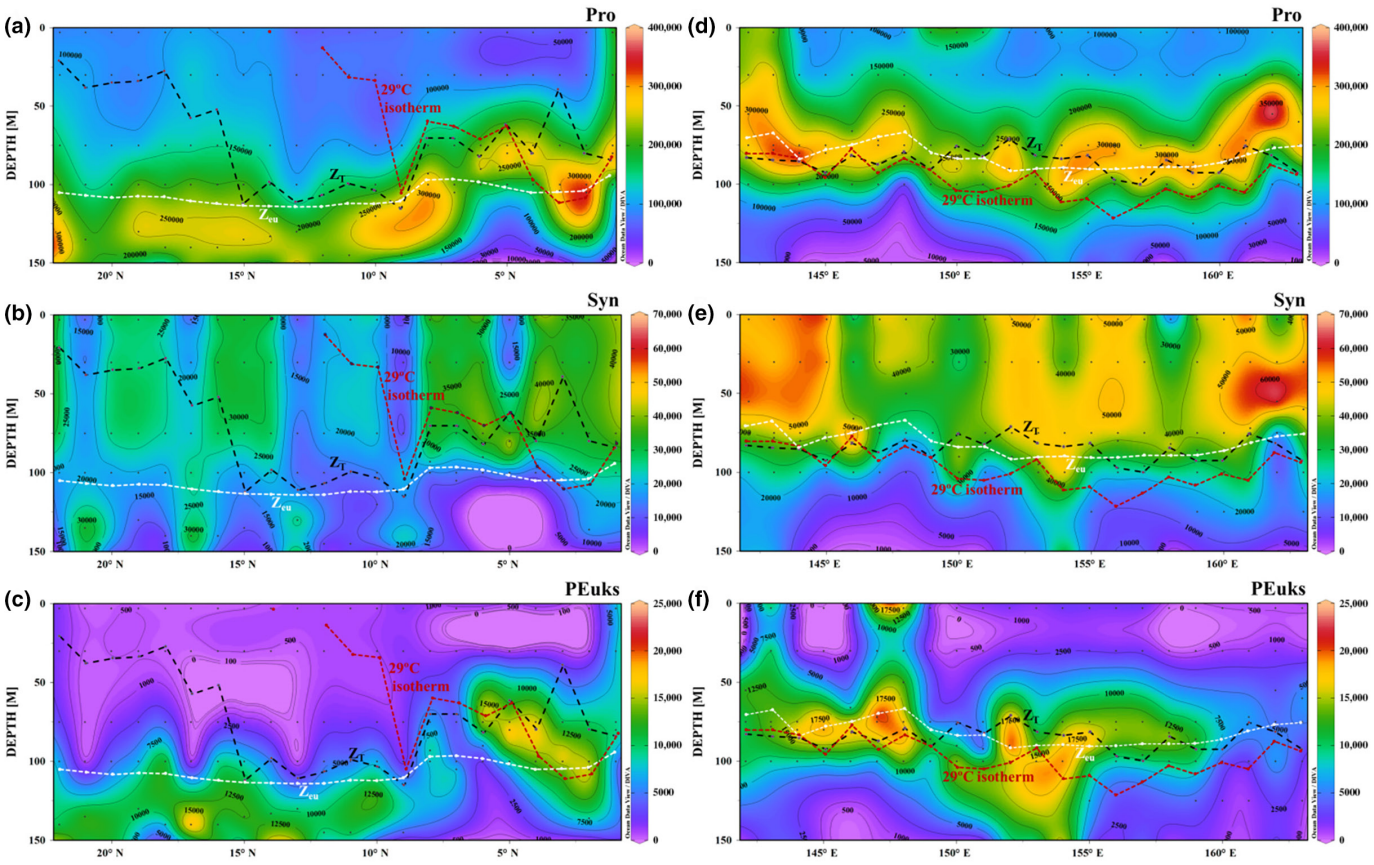
Distributions of Pro, Syn, PEuks abundance (cells/mL) along the (a–c) meridional and (d–f) latitudinal transects. The dashed dark line represents isothermal depth. The dashed white line represents euphotic depth. The dashed dark red line represents the 29°C isotherm. Note the color scales on the right, reflecting the variability in magnitude of different picophytoplankton groups.

Pro abundance varied between 1.73 × 10^2^–3.95 × 10^5^ cells/mL. The highest value along the meridional transect appeared at 100–150 m, except for a 3 m area adjacent to the equator (Figure 7a). The maximum Pro abundance in the latitudinal transect was observed at 75 m, and tended towards 3 and 100 m at the eastern boundary of the warm pool (Figure 7d). Syn abundance was 1.15 × 10^2^–6.54 × 10^4^ cells/mL. A high Syn abundance in the meridional transect appeared above 75 m in the equatorial region (0–8° N) (Figure 7b). Along the latitudinal transect, the vertical distribution of Syn was consistent with temperatures >28.5°C observed beyond 100 m (Figures 4c and 7e). The maximum Syn abundance was detected between 50–75 m. Below 100 m, Syn abundance gradually decreased with depth and was concentrated at 50 m at the east end of the latitudinal transect, and was highest at 30 m at the west end. Furthermore, Syn abundance was slightly higher in the east end than in the west end (Figure 7e). PEuks abundance was 0.64 × 10^2^–2.35 × 10^4^ cells/mL and was lower in the meridional transect (Figure 7c). The maximum PEuks abundance was identified at 75 m in the equatorial region (2–4° N), and the second highest values mainly appeared at 100–150 m. An area of <1000 cells/mL occurred at 150 m adjacent to the equator (0–9° N). The maximum PEuks abundance in the latitudinal transect, found at 75 m, was significantly higher west of 159° E than east of this meridian. Overall, PEuks abundance gradually decreased below 100 m (<1000 cells/mL), but was high at 151–153° E (Figure 7f).

In general, total picophytoplankton abundance varied between 3.85 × 10^2^–4.43 × 10^5^ cells/mL, with Pro being the dominant genus. Syn abundance was approximately five times higher than that of PEuks. The variability in picophytoplankton total abundance (Pro+Syn + PEuks) was consistent with that of the Pro trend. Specifically, Pro was primarily distributed below the isothermal depth (Z_T_), while the subsurface maximum Pro abundance was formed. The maximum Syn abundance tended to be found in nutrient‐rich waters of the Ekman pump‐induced upwelling in the latitudinal region. Vertically, the subsurface maximum PEuks abundance overlapped with the SCM layer, suggesting that PEuks were primary contributors to Chl *a* concentrations.

### 3.5 Picophytoplankton carbon biomass

**FIGURE 8:**
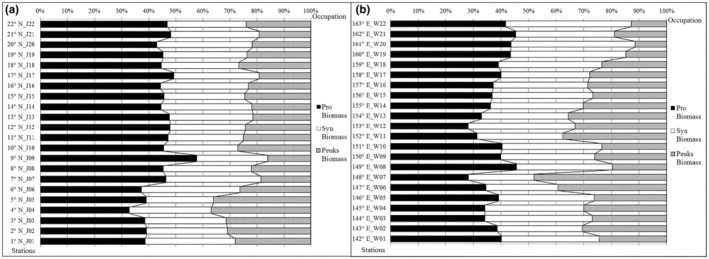
Relative occupations of depth‐integrated (0–150 m) carbon biomass (μgC/L) for picophytoplankton groups along the (a) meridional and (b) latitudinal transects.

The average carbon biomass of total picophytoplankton was slightly higher in the latitudinal transect (13.2 μgC/L) than in the meridional transect (12.0 μgC/L). Pro and Syn accounted for a substantial fraction of the total picophytoplankton biomass (approximately 52%–89%) across the two transects (Figure 8). In contrast, PEuks were not a large component of the total picophytoplankton biomass. The relative biomass of PEuks was low in the meridional transect (24.8%) and slightly increased to 27.0% in the latitudinal transect.

The average proportion of Pro‐carbon biomass in the meridional transect was 44.4% (Figure 8a) with the lowest found at J4 (tropical 4° N); that at J9 in the deep‐sea basin was >57.8%. The proportion of Syn (average 30.8%) had a maximum at J6 (36.5%) and a minimum at J5 (24.9%). Finally, the proportion of PEuks (average 24.8%) was opposite to that of Pro (i.e., largest at J4 and smallest at J9). In the latitudinal transect (Figure 8b), the average proportion of Pro‐carbon biomass was 37.8%, with a maximum at W21 and a minimum at W12. Syn had the next highest proportion (35.2%), with a maximum at W22 and a minimum at W7. In contrast to Syn, PEuks (accounting for 27.0%) had maximum and minimum proportions at W7 and W20, respectively.

### 3.6 Relationship between picophytoplankton and environment

**FIGURE 9:**
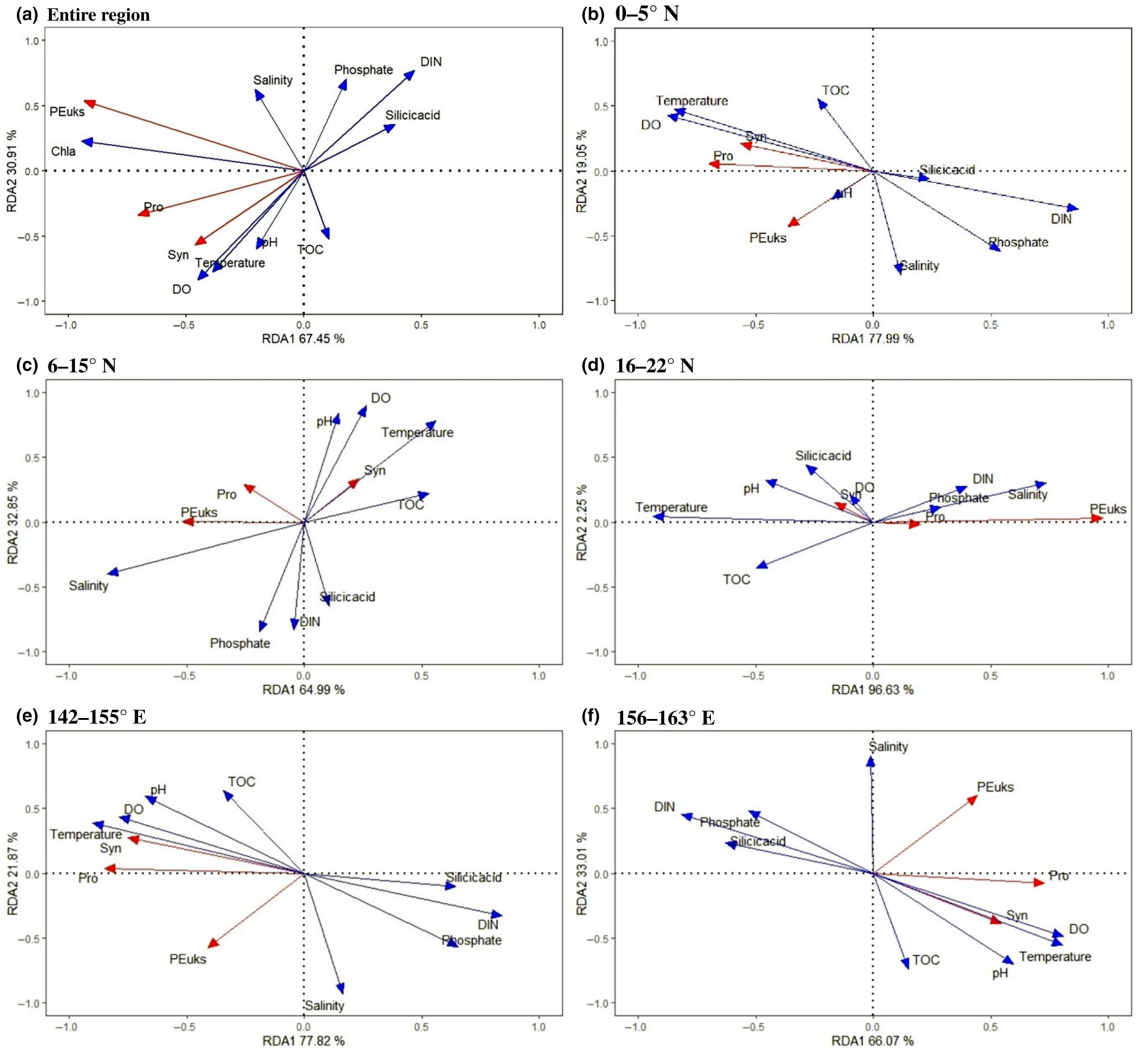
Redundancy analysis (RDA) ordination diagram of the biological communities constrained by oceanographic variables in the tropical western Pacific Ocean (a) and its differentiated diverse physical environments along the (b–d) meridional and (e, f) latitudinal transects. Picophytoplankton abundance was log‐transformed prior to analysis.

In terms of abundances, the three picophytoplankton groups in the two transects were positively correlated with Chl *a*, suggesting that picophytoplankton were major contributors to Chl *a* (Figure 9a–f). Temperature, salinity, DIN, and phosphate (*p* < .01) contributed significantly to the total variance of the survey area (Figure 9a). Pro and Syn were negatively correlated with major nutrients and positively correlated with temperature, and PEuks were positively correlated with salinity. There was a weak positive association between major nutrients and PEuks and between salinity and Pro. This indicated that temperature was the main factor affecting the Pro and Syn distributions, whereas the PEuks distribution was greatly affected by salinity.

Based on the results from this study, the tropical western Pacific was differentiated into diverse physical environments, including Ekman pumping, eddies, and permanent convergence in the WPWP (Figures 2, 3, 4, 5). Consequently, we separated the datasets into three regions for the meridional transect and two regions for the latitudinal transect for correlation analysis (Figure S2). Notably, in the equatorial region of the meridional transect (0–5° N), a considerable negative correlation between nutrients and Pro and Syn was evident (Figure 9b). In the Ekman pumping conditions (6–15° N), Syn and PEuks showed opposite relationships with environmental variables (Figure 9c). In the northern region (16–22° N), hydrological conditions (temperature and salinity) combined with nutrient concentrations were the main regulators of the Pro and Syn distributions (Figure 9d). Whereas, in the latitudinal transect, the Pro and Syn distributions were explained by temperature in the warm pool area (142–155° E) (Figure 9e). At the eastern boundary of the warm pool (156–163° E), PEuks were closely associated with salinity (Figure 9f).

**FIGURE 10:**
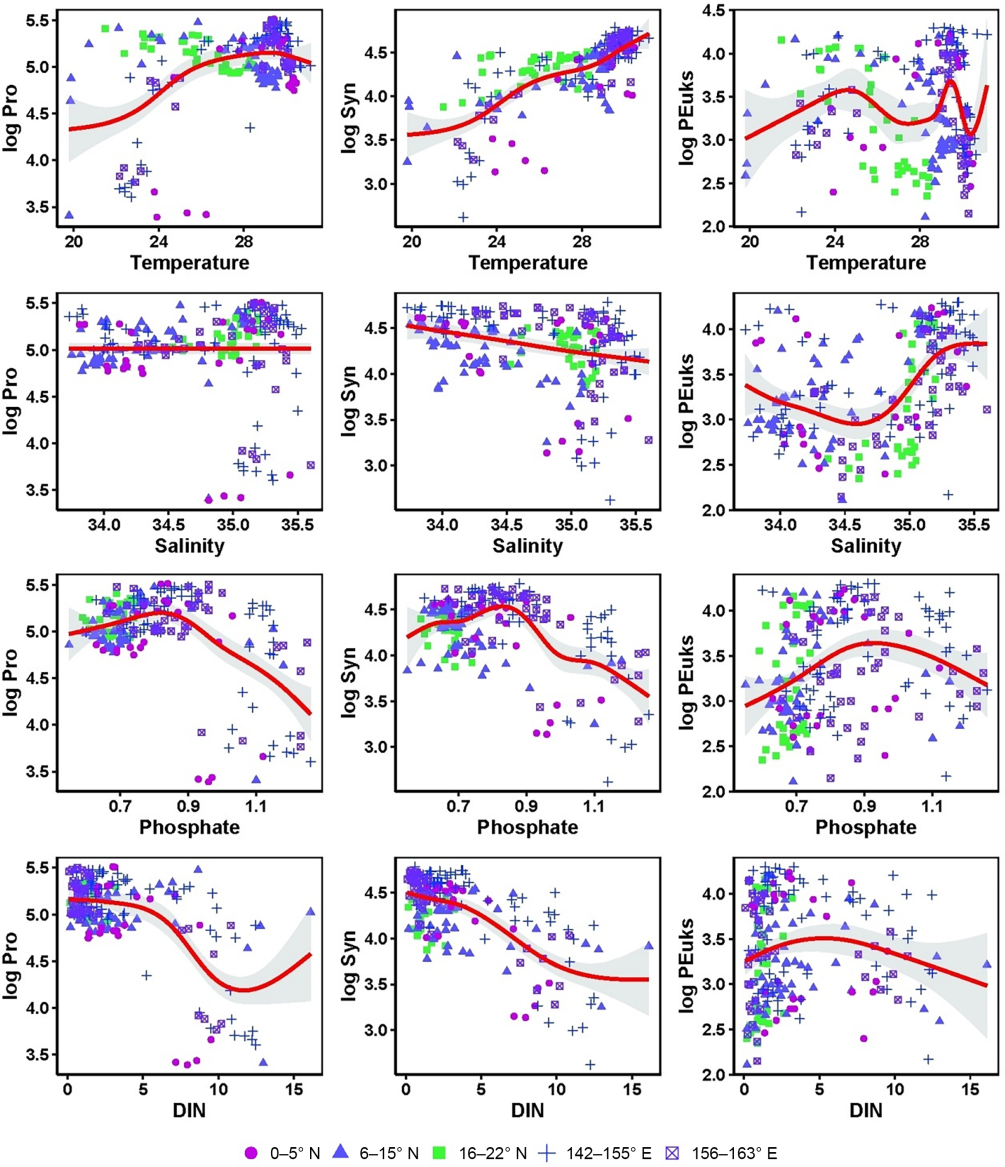
GAMs fitted to the key oceanographic factors and picophytoplankton groups in the western tropical Pacific. Significant relationships (*p* < .05) are indicated as red solid lines, with shaded areas representing the 95% confidence intervals (*R* ^2^ = .639).

GAMs showed that temperature, salinity, DIN, and phosphate were strong predictors of the three principal picophytoplankton groups (Figure 10). In the GAMs, the Syn abundance increased gradually with the increase in temperature, and the Pro abundance tended to be highest at a temperature around 28°C. PEuks exhibited high adaptability to temperature changes. Compared to the trend of PEuks with salinity, the effects of salinity on Pro and Syn were less obvious. All three groups had higher adaptability under low phosphate concentrations. Pro and Syn had higher adaptability under low DIN concentration, and PEuks abundance showed an obvious peak with the increase in DIN. However, Syn abundance decreased rapidly with increasing DIN, and Pro abundance decreased more slowly.

## 4 DISCUSSION

### 4.1 Dominance of *Prochlorococcus* in picophytoplankton community in the tropical western Pacific

The abundances of the three principle picophytoplankton groups determined in this study are comparable to previous results in the oceans, suggesting the major prominence of Pro (Table 2). The picophytoplankton abundance in the tropical western Pacific was similar to that observed in the warm pool, Eastern Equatorial Indian Ocean, Equatorial Pacific, and Seamount in the tropical western Pacific, and slightly lower than that in the western subtropical gyre in the North Pacific, Station ALOHA, and subtropical North Atlantic waters (Table 2). Evidently, Pro forms prevalent picophytoplankton populations in oligotrophic subtropical gyres (Agustí et al., 2019; Bouman et al., 2011; Grob et al., 2007). In terms of biomass, the contribution of Pro in the Pacific Ocean varied from 69.3% in the warm oligotrophic area to 58.7% in equatorial upwelling, with PEuks and Syn accounting for 35% and 7% on average, respectively (Blanchot et al., 2001). The Pro abundance in the present study was much higher than that of Syn and PEuks (the order of magnitude was ×10^5^, ×10^4^, and ×10^3^ cells/mL, respectively) (Figure 7). Moreover, Pro‐carbon biomass was dominant at 7–12° N in the meridional transect and west of 156° E in the latitudinal transect, accounting for 50% and 42% of the total carbon biomass, respectively (Figure 8). In the warm pool, Pro was dominant in terms of cell abundance and estimated carbon biomass (Blanchot et al., 2001). Syn does not colonize deep waters, as does Pro, but has a wider geographical distribution that covers both polar and high‐nutrient waters (Partensky et al., 1999). Previous studies have reported that Syn is usually dominant in mesotrophic or shallow waters (Glover et al., 1986; Partensky et al., 1999). Meanwhile, PEuks are extremely diverse with picophytoplankton taxa distributed widely across several branches of the eukaryotic tree of life (Kuwata et al., 2018; Vaulot et al., 2008). Thus, Syn and PEuks may be abundant in mesotrophic regions, which is consistent with a previous study in the western Pacific Ocean (Zhao et al., 2010).

**TABLE 2:** Comparison in abundance of picophytoplankton principle three groups (×10^3^ cells/mL) in different regions of the global oceans.

Regions	Location	Pro	Syn	PEuks	References
Tropical western Pacific	0–22° N, 142–163° E	0.17–222	0.12–40	0.06–12	This study
Warm Pool	0° N, 167° E	Maximum 190	<1.5 ± 0.1	Maximum 2.3	Blanchot et al. (2001)
Tropical and subtropical western Pacific	2–23° N, 121–130° E	0.12–100	0.15–63	0.03–2.8	Liang et al. (2017)
Seamount in the tropical western Pacific	10.3–10.9° N, 139.9–140.4° E	0.09–153.89 (18.06 ± 30.24)	0.01–1.49 (0.54 ± 0.40)	0.00–3.51 (0.73 ± 0.55)	Zhao et al. (2020)
Equatorial Pacific	0° N, 145° E–160° W	Maximum 180	Maximum 26	Maximum 10	Matsumoto et al. (2004)
Western subtropical gyre in the North Pacific	13.25–33.6° N, 141.5° E	2.05–434	1.94–27.3	0.05–11.5	Girault et al. (2013)
Station ALOHA	22°45′ N, 158° W	140–320	1.1–6.3	0.7–6.2	Campbell et al. (1997)
Eastern equatorial Indian Ocean	10° N–8° S 79° E–98° E	0.32–225	0.01–35	0.01–13	Wei et al. (2019)
Subtropical North Atlantic	5° S–24° N	183 ± 111	9.3 ± 13	1.2 ± 1.9	Buck et al. (1996)

The ratio of cell abundance of Pro to Syn is used to emphasize the relative importance of Pro. The typical Pro:Syn ratio in oligotrophic waters ranges 50–200 (Campbell et al., 1998). The abundance ratio of Pro and Syn in this study was between 1.17 and 39.76 among all samples, with average depth‐integrated Pro:Syn ratios of 8.29 and 5.53 in the latitudinal and meridional transects, respectively (Table 1). Similar results were also seen in the south of the South China Sea (5.44–43.20) by Wang, Tan, Huang, Ke, Tan, et al. (2016); Wang, Tan, Huang, Ke, and Zhou (2016), suggesting no nutrient deficiency in the study area during the sampling period. The reason was that the mixing of water in the tropical western Pacific was strengthened in spring from April–May, resulting in the Ekman pumping‐induced upwelling of nutrients by 100 m (6–15° N). Alternatively, occasional phenomena may occur (such as mesoscale eddies, including cyclonic eddies at 141° E, 6–9° N, and anticyclonic eddies at 143° E, 19–21° N), increasing nutrients in local regions. Overall, the tropical western Pacific was generally typical high‐temperature oligotrophic water, but nutrient concentrations increased locally. In general, Pro dominate in the oligotrophic environments of subtropical and tropical oceans, but their distributions are limited at high latitudes by low temperatures (Johnson et al., 2006; Partensky et al., 1999) and are also limited in coastal waters, upwelling areas, and temperate oceans by environmental factors such as nutrient availability (Berube et al., 2015; Partensky & Garczarek, 2010). Low temperature has a significant limiting effect on the Pro distribution (Johnson et al., 2006). Although the limited effects of high nutrient concentrations in coastal regions or upwelling zones could constrain Pro‐growth, an increase in nutrient availability in oligotrophic systems can appreciably elevate the Pro abundance (Baer et al., 2017; Johnson et al., 2006; Wei et al., 2019, 2022). We speculated that the reason for the dominance of Pro was its environmental adaptability, and the differentiation of Pro ecotypes and sub‐ecotypes reflected its continual adaptation to the changeable marine environment (Yan et al., 2020). Malmstrom et al. (2010) similarly observed that each Pro ecotype responds differently to variations in light. Therefore, in the present study, Pro was dominant in the tropical western Pacific due to its demonstrated ecological plasticity in terms of nutrient and light requirements (Figure S2).

### 4.2 Physical processes regulating spatial variability in picophytoplankton abundance in the tropical western Pacific

The north‐easterly and south‐easterly trade winds prevail in the tropical western Pacific (Hou et al., 2014). These winds affect phytoplankton biomass by influencing wind‐driven currents and nutrient‐rich deep water upwelling through Ekman pumping (Hou et al., 2014; Martin & Richards, 2001). In the western Pacific, Ekman pumping usually occurs mainly in the sea area west of 160° E and south of 15° N. A small upward motion of the thermocline induced by Ekman pumping was recorded in the 7–9° N region (Delcroix & Henin, 1989; Tournier, 1989). Under Ekman pumping action, the low‐temperature and high‐nutrient North Pacific intermediate water can surge to the subsurface at around 7–9° N, providing a nutrient basis for massive phytoplankton proliferation. This is a key process that affects not only the water column stability but also the source and distribution of restricted nutrients in the upper euphotic zone of the western Pacific (Dugdale & Goering, 1967; Hu et al., 2015; Stewart, 2004). During the present study, the SLA and hydrographic conditions at 140–145° E and 6–9° N were consistent with the presence of an upwelling induced by Ekman pumping (Figure 2). The direct relationship between Pro and water column stability, rather than temperature and nutrient, would explain the high Pro abundances in the oligotrophic waters and its absence in polar regions where stratification is seasonal or episodic (Bouman et al., 2011; Partensky et al., 1999; Zubkov et al., 1998). Higher Pro abundances have been observed in more stratified waters, whereas Syn and PEuks are more abundant when mixing prevails (Blanchot & Rodier, 1996; Bouman et al., 2011). Thus, in this study, Pro abundance at 100–150 m was relatively low by 6–9° N due to the presence of relatively unstratified layers resulting from Ekman pumping. However, PEuks and Syn abundances did not increase significantly at the same depth, with strong mixing of Ekman pumping‐induced upwelling waters and high nitrate concentrations (>16.06 μmol/L) (6–15° N) (Figures 4 and 7). However, nutrients did not significantly explain the distribution of abundance of the three principle picophytoplankton groups at 6–15° N (Figure 9d and 10). It is reasonable to infer that the physical characteristics of low‐temperature upwelling waters combined with nutrient enrichment were the main regulators of picophytoplankton abundance in this area (6–9° N).

Process model studies also indicate that both vertical and lateral nutrients supplied by mesoscale eddies facilitate strong picophytoplankton growth near the margins of an oligotrophic gyre (Oschlies, 2002). Eddy‐related nutrient injections within the euphotic zone, particularly cyclonic eddies, can significantly enhance biological production in oligotrophic waters (McGillicuddy & Jr Robinson, 1997, Vaillancourt et al., 2003). In the present study, the SLA and hydrographic conditions at 143° E, 22–24° N were consistent with the presence of a cyclonic eddy, and with an anticyclonic eddy at 143° E, 19–21° N (Figure 2). Correspondingly, the depth‐integrated maximum Pro abundance was found at J22 (22° N, edge of the cyclonic eddy), which was higher than that at J2 in the warm equatorial area (high temperature), and at J21 (21° N), J20 (20° N), and J19 (19° N) in the adjacent anticyclonic eddy (Figures 2 and 7). During the sampling period, J22 (at the edge of the cyclonic eddy; Figure 2) showed a lower nutrient content and temperature (Figure 3 and 4) than J21 (at the edge of the anticyclonic eddy; Figure 2). These conditions were closely associated with the increase in Pro abundance at 100–150 m (Figure 7a). It appeared that the cyclonic eddy edge could increase the Pro abundance, whereas the anticyclonic eddy edge decreased the Pro abundance (Ning et al., 2008; Wang, Tan, Huang, Ke, Tan, et al., 2016; Wang, Tan, Huang, Ke, & Zhou, 2016). Additionally, Pro was absent from the anticyclonic eddy core and edge, with Syn primarily distributed outside the anticyclonic eddy (J17, J16, and J15) (e.g., Rodríguez et al., 2003). Moreover, DIN and phosphate explained the significant Pro abundance distribution at 16–22° N (Figures 9d and 10); hence, we inferred that nutrient availability, as a consequence of the movement of cyclonic eddies, may have promoted the high Pro abundance at 100–150 m at J22 (the edge of a cyclonic eddy) (Mahadevan et al., 2008; Šantić et al., 2011).

In addition, it has been documented that Syn populations increase in warm nutrient‐enriched coastal waters (Partensky et al., 1999; Phlips et al., 1999) and areas influenced by upwelling, as reported for a north‐eastern African upwelling (Alonso‐Laita & Agustí, 2006) and other continental areas (Blanchot et al., 2001; Ribeiro et al., 2016; Zubkov et al., 1998). This response to regionally increased nutrient availability has also been reported in the PEuks group (Blanchot et al., 2001; Ribeiro et al., 2016; Yuan et al., 2020; Zubkov et al., 1998). However, in this study, adjacent to a cyclonic eddy at 141° E, 6–9° N, depth‐integrated minimum abundances of Syn and PEuks were found consistently at J9 (143° E, 9° N; edge of the cyclonic eddy) with high temperatures and high nutrients beyond 100 m (Figure 4). The vertical distributions of Syn and PEuks abundance above 100 m at J9 were also considerably lower than those at adjacent stations J8 and J10 (outside the cyclonic eddy; Figure 7b,c). This result is consistent with observations in the subtropical North Pacific where Syn and PEuks were 3.6‐fold more numerically abundant outside the cyclonic eddy than inside (Vaillancourt et al., 2003). Moreover, the maximum PEuks abundances were found at 145 m on the outside of the cyclonic eddy and were lifted to 100 m at its edge (e.g., Kang et al., 2022). These effects are explained in the case of a cyclonic eddy based on the doming of its isopycnals and nutricline (Lévy et al., 2009; Ning et al., 2008). In general, the eddies shaped different patterns of picophytoplankton communities compared to those in other water systems.

Ocean fronts are important and ubiquitous dynamic features and sites of high biological productivity (Clayton et al., 2014; Yoder et al., 1994). In the tropical western Pacific, the sea level is higher in the west and lower in the east and is strongly correlated with Chl *a* (Olita et al., 2011; Wilson & Adamec, 2001). During this study, the picophytoplankton abundance, carbon biomass, and Chl *a* concentration were higher in the west than in the east from 155° E eastward along the equator in the latitudinal transect. Picault et al. (1996) documented a permanent convergence and obvious salinity front in the surface water at the eastern boundary around 155° E of the WPWP, which leads to the deviation of nutrient concentrations on both sides of the front. This front is the result of the intersection of a westward diffusion flow of low‐temperature and high‐salinity water in the central and eastern equatorial Pacific and an east–west diffusion flow of high‐temperature and low‐salinity water in the western equatorial Pacific, which is controlled by westerly winds (Flament et al., 1996; Picault et al., 1996). Furthermore, under the influence of the El Niño‐Southern Oscillation, the position of the front changes interannually by >50 longitudes, moving eastward during El Niño and westward during La Niña. Water convergence and the front aggravate plankton and nekton accumulation (Picault et al., 1996; Mcphaden et al., 2006; Baltar & Arístegui, 2017). For example, observations across the Kuroshio Extension Front revealed a sharp divide between clearly distinct phytoplankton communities on either side of the front, suggesting an ecological barrier, and that the front drove lateral nutrient transport southwards into the subtropical gyre with elevations in the phytoplankton biomass (Clayton et al., 2014).

### 4.3 Correspondence of picophytoplankton distribution with particular reference to temperature, light irradiance, and nutrient concentration in the tropical western Pacific

Pro prefers high temperatures and has an upper temperature limit of 30°C and a lower limit of 10°C (Moore et al., 1995). Temperature often limits Pro to subtropical and tropical waters (Chisholm et al., 1988; Follows et al., 2007), and its abundance has a relatively large positive response to an increase in temperature (Flombaum et al., 2013; Johnson et al., 2006). As the temperature increased along the meridional transect from north to south, there was an increase in the abundance of all three picophytoplankton groups, suggesting that temperature was an important regulating factor, especially when the temperature exceeded 28°C at 5° N (Figure 10). The temperature above 150 m in the latitudinal transect was 23.5–30.3°C, where Pro was primarily distributed. Syn is reportedly more sensitive to temperature changes, and optimal growth occurs at >19°C; hence, its abundance changes seasonally (Chang et al., 1996). During this study, Syn was found in patches along the meridional transect and was continuously distributed in waters with temperatures >28.5°C in the latitudinal transect (Figure 10). Due to their high species richness and adaptation to extreme environments, PEuks are distributed more widely than Pro and Syn (Virginia et al., 2002). PEuks were detected in each layer of each station and in both transects in this study, usually forming a subsurface maximum abundance, and corresponding to SCM.

**FIGURE 11:**
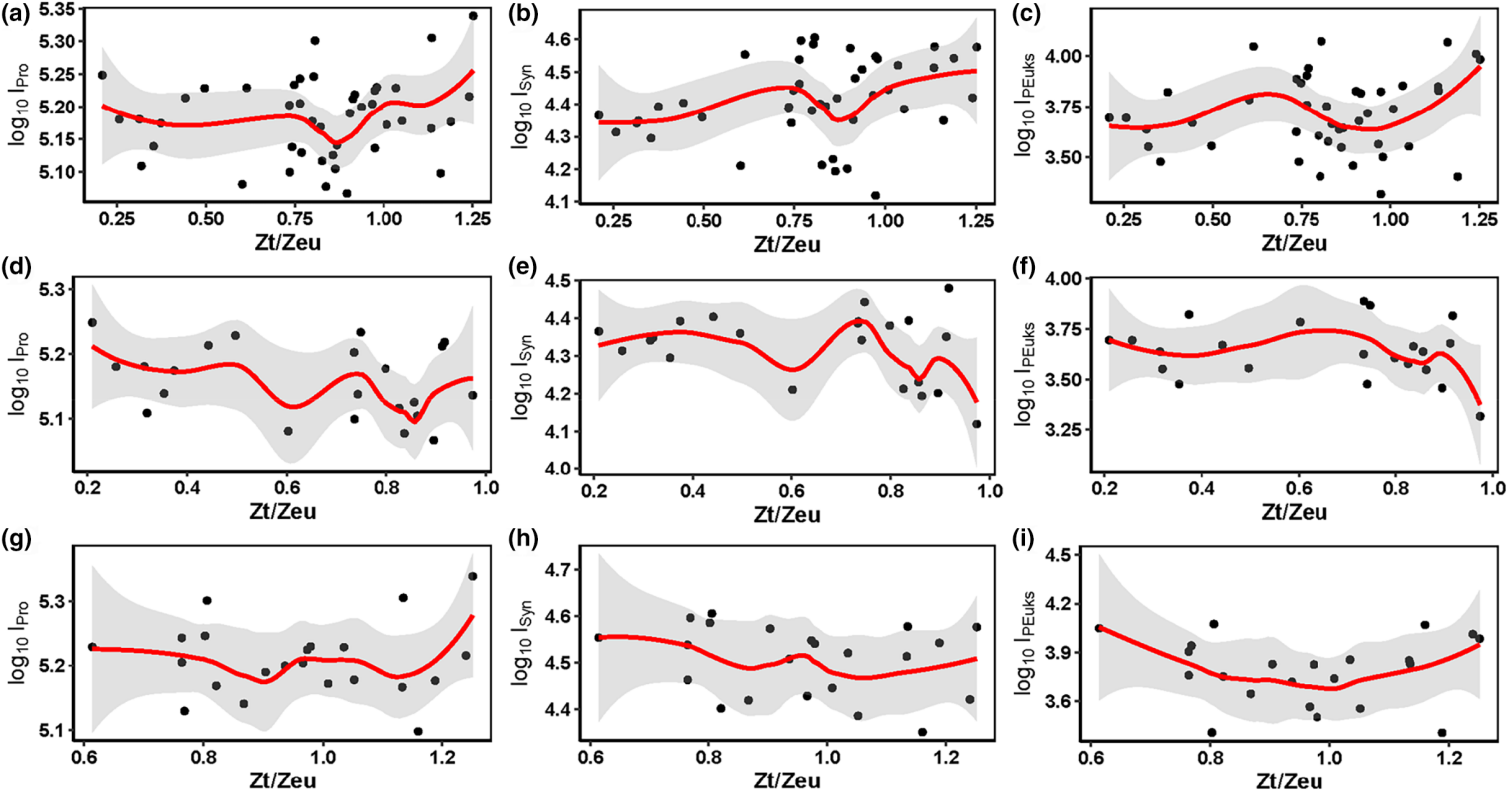
Relationships between depth‐integrated (0–150 m) abundances (cells/mL) of picophytoplankton groups and the isothermal depth (Z_T_) divided by the euphotic depth (Z_eu_) in the study area (a–c) and its diverse physical environments along the (d–f) meridional and (g–i) latitudinal transects. Solid lines represent local polynomial regression (loess) fitting lines, with shaded areas representing the 95% confidence intervals.

Light irradiance is a key limiting factor for the spatiotemporal picophytoplankton distribution (Agustí et al., 2019; Flombaum et al., 2013). Owing to the high sunlight intensity on the surface, maximum picophytoplankton abundance usually occurs in the subsurface (Cai et al., 2007; Marchant et al., 1987; Noh et al., 2006). It has also been reported that the subsurface maximum of PEuks abundance appears at the bottom of the euphotic zone (Cai et al., 2007). In addition, different picophytoplankton groups have different light adaptations, and thus different niches (Coello‐Camba & Agustí, 2021; Flombaum et al., 2020; Moore et al., 2002; Takahashi & Hori, 1984). Pro has higher adaptability to low light than Syn (Agustí & Sanchez, 2002). Syn usually inhabits the upper euphotic layer, whereas Pro can still be found at 200 m (Cai et al., 2007). Previous studies have suggested that Pro is more sensitive than Syn to sunlight, particularly ultraviolet (Llabrés & Agustí, 2006); however, this has not been demonstrated for Syn. As the light wavelength decreases from red to blue light, so does the ability of light to penetrate water. Accordingly, Pro is better adapted to capture blue wavelengths that predominate deeper in the water column (Dusenberry et al., 2000; Grébert et al., 2018; Moore et al., 1995). In the weaker mixed water in the western Pacific, the Syn abundance decreased compared to that on the surface (Liu et al., 2002; Moon‐van der Staay et al., 2001). This explained why Syn was concentrated in the upper layer at 100 m beyond 9–13° N in the meridional transect during this study, which was more obvious in the latitudinal transect. In addition, the vertical picophytoplankton distribution is not dependent on the position of the seasonal thermocline in the subtropical Pacific (Semina et al., 2000). The strength of the Z_T_ (isothermal depth) separated two contrasting environments with different resource limitations (nutrients versus light) that modulated the picophytoplankton community structure. With respect to Z_T_/Z_eu_, the I_Pro_, I_Syn_, and I_PEuks_ decreased monotonically with Z_T_/Z_eu_ in both transects (Figure 11). The maximum Syn abundance was centered at Z_T_/Z_eu_ values of 0.7–0.9 along the meridional transect, and those of Pro and PEuks were centered at lower Z_T_/Z_eu_ values of 0.6–0.7. At Z_T_/Z_eu_ values of 1.0–1.2 along the latitudinal transect, Syn abundance was strongly reduced, and only Pro and PEuks thrived under such low light conditions. This agrees with previous results from oligotrophic oceans (Agustí et al., 2019; Partensky et al., 1999).

**FIGURE 12:**
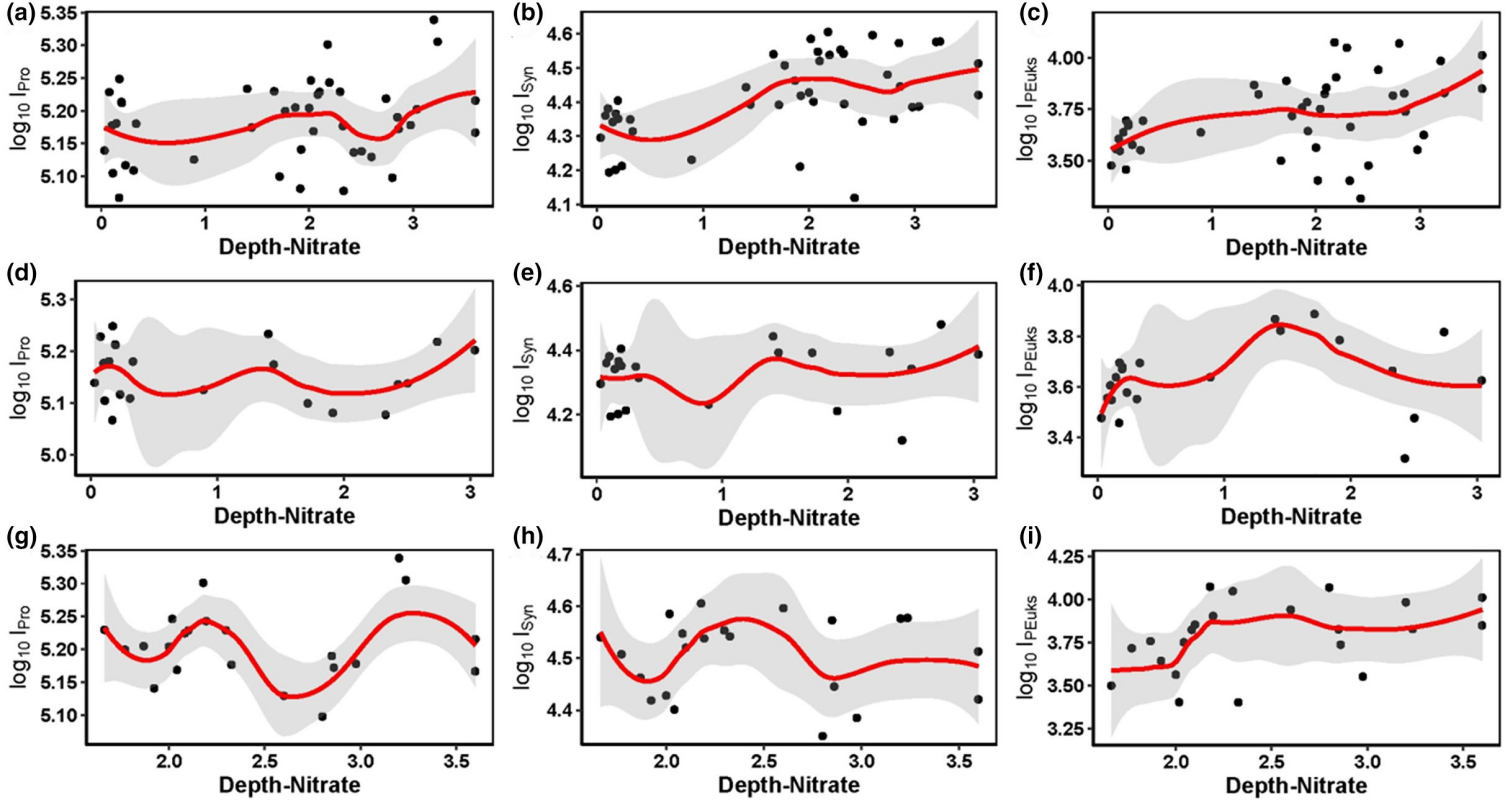
Relationships between depth‐integrated (0–150 m) abundances (cells/mL) of picophytoplankton groups and depth‐integrated nitrate concentrations in the study area (a–c) and its diverse physical environments along the (d–f) meridional and (g–i) latitudinal transects. Solid lines represent local polynomial regression (loess) fitting lines, with shaded areas representing the 95% confidence intervals.

The univariate responses of picophytoplankton to increased nutrient concentrations in the tropical western Pacific obtained here were complicated, in contrast with the global oligotrophic ocean where increased nutrients led to negative correlations with Pro but positive correlations with Syn and PEuks (Agustí et al., 2019). During the present study, Pro and Syn were negatively correlated with the nutrient concentrations, whereas PEuks were positively correlated (Figures 9a and 10). The same conclusion was reached by Gaeta et al. (1999) and Brandini et al. (2019), who found a negative correlation between Pro abundance and nutrient concentrations. However, comparisons with other observations indicate that nutrient availability may be a major factor responsible for the oceanic distribution of Syn and PEuks (Agawin et al., 2000; Agustí et al., 2019; Partensky et al., 1999; Zhao et al., 2010). In particular, the nitrate concentration is important for modulating Pro abundance across the eastern South Pacific Ocean (Grob et al., 2007). In the pooled dataset of the present study, weak decreases with depth‐integrated nitrate were found for I_Pro_ rather than an increase in I_Syn_ and I_PEuks_ in the study area (Figure 12a–c). The same trend was shown in the meridional transect (Figure 12d–f). In the latitudinal transect, weak decreases with depth‐integrated nitrate were found for I_Pro_ and I_Syn_; whereas, weak increases with depth‐integrated nitrate were found for I_PEuks_ (Figure 12g–i). Hence, the widespread presence of Pro and the intermittent presence of PEuks and Syn in elevated nutrient environments in the tropical western Pacific suggested that the linkage between abundance and nutrient availability is more complex for this lineage than previously understood.

## 5 CONCLUSIONS

In this study, we documented the large‐scale distributional patterns of the picophytoplankton assemblage composition, abundance, biomass, and associated oceanography in the tropical western Pacific. The diverse physical environments, including eddies, Ekman pumping, and frontal interaction, significantly influenced the picophytoplankton distribution, causing dramatic changes in the spatial distribution patterns. Analyses of the picophytoplankton abundance and biomass showed that Pro was predominant in both latitudinal and meridional transects, followed by Syn and PEuks. In contrast to other studies, we showed that PEuks were not major contributors to the picophytoplankton biomass. Overall, Syn and PEuks showed opposite relationships concerning oceanographic variables. Nutrient availability had a two‐faceted role in regulating the spatial patterns of picophytoplankton in diverse latitudinal and meridional environments. In particular, water temperature and light irradiance were key determinants of the spatial variability of picophytoplankton in the tropical western Pacific. Overall, our findings provide valuable insights into the interaction between picophytoplankton communities and their ecological mechanism.

## AUTHOR CONTRIBUTIONS

**Yu Wang:** Conceptualization (lead); data curation (lead); funding acquisition (equal); investigation (equal); methodology (equal); writing – original draft (lead). **Feng Zhao:** Conceptualization (equal); funding acquisition (equal); supervision (equal); validation (equal). **Xuebao He:** Conceptualization (equal); formal analysis (equal); project administration (equal); visualization (equal). **Weibo Wang:** Formal analysis (equal); investigation (equal). **Lin Chang:** Formal analysis (equal); investigation (equal). **Jianhua Kang:** Conceptualization (equal); data curation (equal); project administration (equal); visualization (equal).

## CONFLICT OF INTEREST STATEMENT

The authors declare that the research was conducted in the absence of any commercial or financial relationships that could be construed as a potential conflict of interest.

## Supporting information


Data S1.

**Figure S1.** **–S2.**

## Data Availability

The data that support the findings of this study are uploaded as a Supplementary Excel File. Meanwhile, the data are treated as Private for peer review in Dryad at https://doi.org/10.5061/dryad.b8gtht7j3, since the related article has not been accepted.
