# Supplementary material for: Latitudinal and meridional patterns of picophytoplankton variability are contrastingly associated with Ekman pumping and the warm pool in the tropical western Pacific
Source: Ecol Evol. 2023 Oct 19;13(10):e10589. doi: 10.1002/ece3.10589 (PMC10587655; doi:10.1002/ece3.10589)
Supplement: Supplementary file 2 — Figure S1. –S2. [file ECE3-13-e10589-s001.docx]

Supplementary Materials for

**Latitudinal and meridional patterns of picophytoplankton variability are contrastingly associated with Ekman pumping and warm pool in the tropical western Pacific**

Yu Wang^1#^, Feng Zhao^2#^, Xuebao He^1^, Weibo Wang^1^, Lin Chang^1^, Jianhua Kang^1*^

^1^Third Institute of Oceanography, Ministry of Natural Resources, Xiamen 361005, PR China

^2^Institute of Oceanology, Chinese Academy of Sciences, Qingdao 266071, PR China


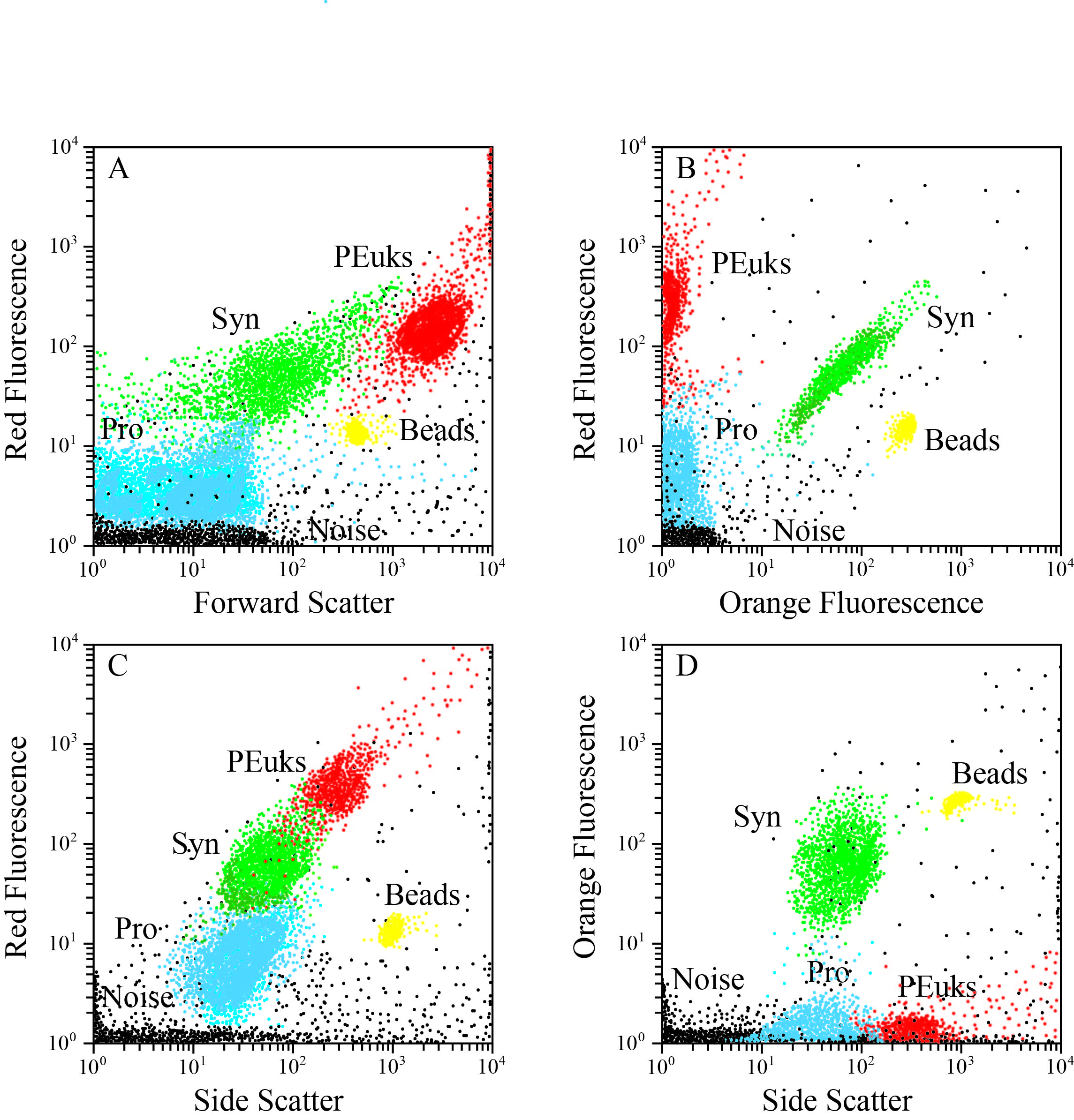


**Fig. S1** A cytogram from the flow cytometer showing how picophytoplankton cells were counted. Example of the water sample was collected at 50 m of the Station W2 (143°E, 0°N) in the equator of the western Pacific Ocean. The abundance, N(cell/mL), for each picophytoplankton population in a field sample is calculated from the equation: N=C/(T×R)×CF×1000μL/mL. Where C is the number of events acquired (cells) for a specified population, T is the duration of analysis (min), R is the sample delivery rate (μL/min), and CF is the correction factor to account for sample dilution owing to preservation, bead addition, or staining.


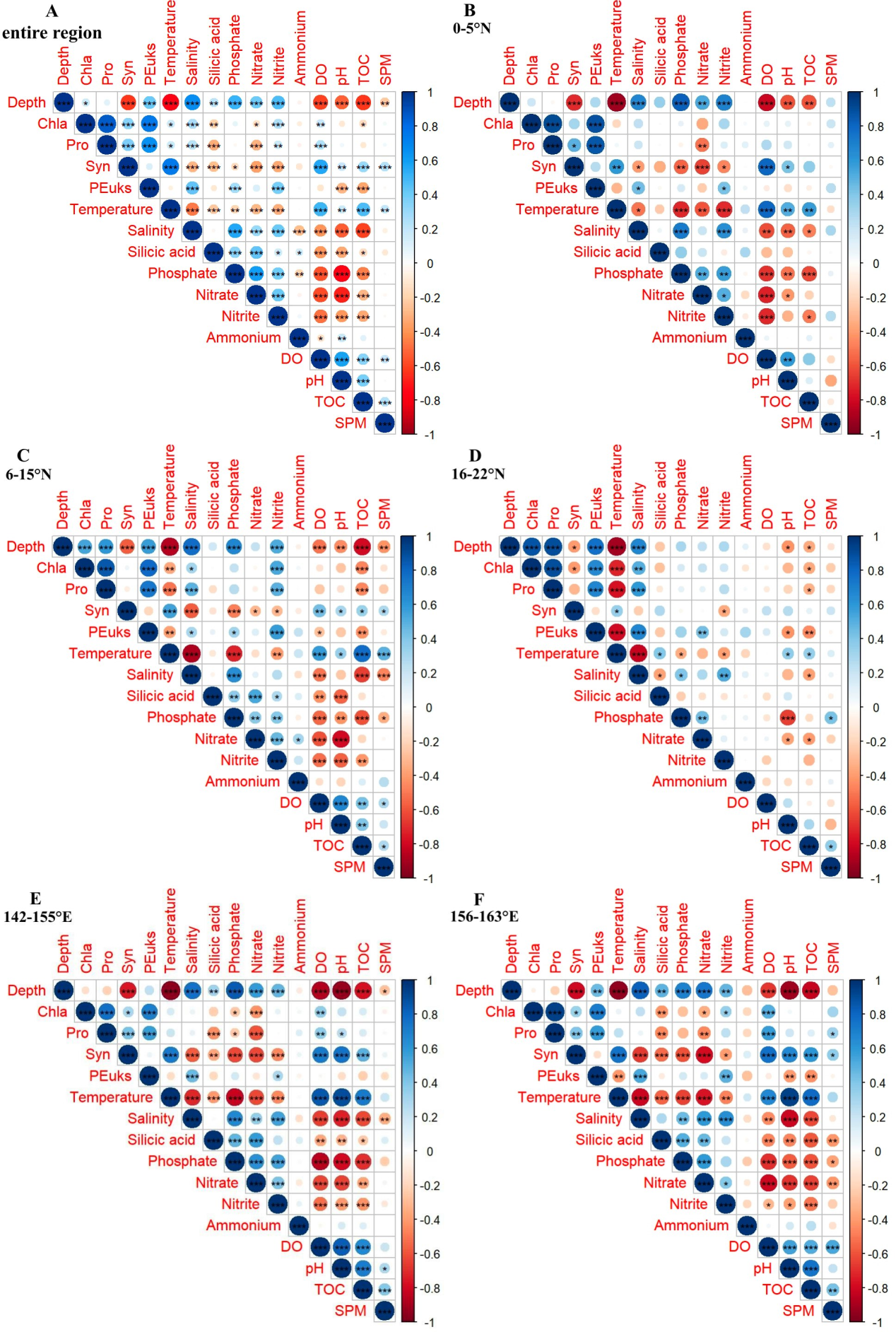


**Fig. S2** The Spearman’s correlation coefficient matrix between picophytoplankton abundances and oceanographic variables in the tropical western Pacific Ocean (A) and its diverse physical environments along the (B, C, D) meridional and (E, F) latitudinal transects. Red represented negative correlations, and blue represented positive correlations. The size of circle indicated the strength of correlation. ***P <0.001; **P <0.01; *P<0.05.
